# Adipokines, inflammation, insulin resistance, and carotid atherosclerosis in patients with rheumatoid arthritis

**DOI:** 10.1186/ar4384

**Published:** 2013-11-19

**Authors:** Yoon Kang, Hee-Jin Park, Mi-I Kang, Hyang-Sun Lee, Sang-Won Lee, Soo-Kon Lee, Yong-Beom Park

**Affiliations:** 1 Division of Rheumatology, Department of Internal Medicine, Yonsei University College of Medicine, 50 Yonsei-ro, Seodaemun-gu, 120-752, Seoul, Republic of Korea

## Abstract

**Introduction:**

Cardiovascular (CV) morbidity and mortality are increased in patients with rheumatoid arthritis (RA). Inflammation is thought to be an important factor in accelerated atherosclerosis in RA, whereas insulin resistance is a known risk factor for atherosclerosis in RA. We hypothesised that adipokines could be a link between inflammation, insulin resistance, and atherosclerosis in RA.

**Methods:**

The common carotid artery (CCA) intima-media thickness (IMT), CCA resistive index (RI), and carotid plaques were measured by ultrasonography in 192 patients with RA. Insulin resistance was assessed by the homeostasis model assessment for insulin resistance (HOMA-IR). Serum adiponectin, leptin, resistin, tumor necrosis factor-α, and interleukin (IL)-6 concentrations were determined.

**Results:**

The CCA RI was associated with CCA IMT and the estimated total plaque volume after adjustment for conventional CV risk factors. Among adipokines, resistin and IL-6 were correlated with inflammatory parameters. Leptin and leptin:adiponectin (L:A) ratio were correlated with metabolic risk factors, including HOMA-IR. And L:A ratio was related to the CCA RI after adjustment for conventional and nonconventional CV risk factors, including HOMA-IR, erythrocyte sedimentation rate and C-reactive protein.

**Conclusion:**

L:A ratio was associated with HOMA-IR and carotid RI. L:A ratio might be an independent factor for predicting cardiovascular risk in patients with RA.

## Introduction

Cardiovascular (CV) morbidity and mortality are increased in patients with rheumatoid arthritis (RA) [1-3]. However, the pathogenesis of accelerated atherosclerosis in RA is not completely understood, and cannot be explained solely by conventional CV risk factors such as age, male sex, body mass index (BMI), smoking, lipid profile, hypertension and diabetes mellitus [2,4]. Inflammation is an important nonconventional factor in the increased CV risk observed in RA [5,6]. Insulin resistance, which is also important in the development and progression of atherosclerotic CV disease [7], has been suggested as a risk factor for atherosclerosis in RA [8,9]. Insulin resistance is linked to obesity, especially visceral fat accumulation, which alters adipokine secretion profiles, and is recognized as a state of adipose tissue dysfunction. On the other hand, insulin resistance contributes to increased systemic inflammation by altering adipokine secretion, which leads to activation of proinflammatory signaling pathways [10,11]. Adipokines include adiponectin, leptin, resistin, and some proinflammatory cytokines, such as tumor necrosis factor alpha and interleukin (IL)-6. However, the relationship between adipokine concentrations, inflammation, insulin resistance, and atherosclerosis in patients with RA has not been clearly defined.

The common carotid artery (CCA) intima-media thickness (IMT) had been established as a valid marker of early atherosclerosis [12]. Similarly, the carotid resistive index (RI) correlates with the severity of atherosclerosis [13]. The IMT is a morphological parameter and represents the histologically verified intima-media segment of the vascular wall. In contrast, the RI is a hemodynamic parameter based on Doppler technology and relates to vascular resistance [13]. Whereas the carotid IMT has been reported in patients with RA in many studies [14], the RI in these patients has not yet been studied.

We assessed the relationship between adipokines, inflammation, insulin resistance and carotid atherosclerosis via CCA IMT, plaque and RI to test the hypothesis that serum adipokines are a link between inflammation, insulin resistance and atherosclerosis in patients with RA.

## Methods

### Study population

One hundred and ninety-two consecutive patients who were diagnosed with RA at the rheumatology clinic in Severance Hospital, Yonsei University Health System, Seoul, Korea were recruited between September 2011 and January 2012. All patients fulfilled the American College of Rheumatology 1987 revised criteria for classifying RA [15]. Patients with diabetes mellitus were excluded. The study was approved by the Institutional Review Board of Severance Hospital, and patients gave their written informed consent.

### Clinical assessments

Clinical characteristics were assessed on the same day as the carotid ultrasound was performed. Clinical information, including age, sex, and disease duration, history of hypertension, medications, and smoking were assessed by questionnaire. Medications for treatment of RA, including biologics, were assessed by their prescription. Height, body weight, waist circumference, and blood pressure were measured. Tender and swollen joint counts were counted by the same rheumatologist. Disease activities of patients were assessed by the patients’ statements of global well-being (via 100 mm visual analogue scale) and the 28-joint disease activity (via the DAS28 ESR calculator using the erythrocyte sedimentation rate (ESR)).

### Laboratory assessments

Fasting serum insulin, glucose, high-density lipoprotein (HDL) cholesterol, low-density lipoprotein cholesterol, triglycerides (TG), ESR, and C-reactive protein (CRP) concentrations were determined in the hospital clinical laboratory. The homeostatic model assessment for insulin resistance (HOMA-IR) index was calculated. Serum concentrations of adiponectin, leptin, resistin, tumor necrosis factor alpha, and IL-6 were measured using the Lincoplex Milliplex immunoassay (Merck Millipore, Billerica, MA, USA).

### Carotid ultrasound

Both carotid arteries were evaluated with high-resolution ultrasonography (HD15; Philips, Bothwell, WA, USA) with a L12-5 transducer. Three different longitudinal views (anterior oblique, lateral, and posterior oblique) and transverse views of both carotid arteries were obtained. For IMT measurements, far walls of both the right and the left common carotid arteries 2 cm downstream from the bifurcation were imaged at the lateral view. QLAB’s IMT-quantification software measurement plug-in (Philips Healthcare, DA Best, the Netherlands) was used to increase the consistency and reliability of IMT measurements. The presence of carotid plaque was investigated in the internal carotid artery, the external carotid artery, the bulb and the CCA. The plaque was identified as a discrete projection of ≥50% from the adjacent wall into the vessel lumen. The height and length of a plaque were measured in the longitudinal view, and its width was measured in the cross-sectional view. Plaque volume was estimated automatically by the program plug-in after measuring each length of the three-dimensional axis of the plaque. For RI measurements, a pulsed wave-flow spectrum was recorded and frozen from both middle/distal common carotid arteries after 5 seconds of multiple identical waveforms. The RI was calculated automatically according to the Pourcelot formula as follows [16]:

RI = (peak systolic velocity – end-diastolic velocity)/peak systolic velocity

A highly skilled single sonographer, blinded to the subjects’ characteristics, performed all examinations throughout the study.

### Statistical analyses

Data were expressed as the median with interquartile range for continuous variables. For categorical variables, counts and percentages were calculated. The relationships between variables were determined by Spearman’s correlation analysis because variables were not normally distributed. Multiple linear regression was used to analyze the RI and adjust the confounders: conventional CV risk factors included age, sex, smoking, systolic blood pressure, BMI, waist circumference, glucose, TG, HDL cholesterol and low-density lipoprotein cholesterol; and nonconventional CV risk factors included inflammatory markers such as ESR and CRP. Statistical significance was considered *P* <0.05. All statistical analyses were carried out using SPSS software (version 19.0; SPSS Inc., Chicago, IL, USA).

## Results

### Characteristics of patients with RA

One hundred and seventy-six patients (91.7%) were women. The median age of patients was 55 years (45 to 62 years). Median disease duration was 9 years (4 to 15 years), median CRP was 1.7 mg/l (0.5 to 5.5 mg/l) and median DAS28 ESR was 3.1 (2.5 to 4.1), and most patients in this study had low disease activities of RA. Median BMI was 22.3 kg/m^2^ (20.2 to 24.6 kg/m^2^) and the median HOMA-IR was 1.46 (0.96 to 2.27). Other demographic characteristics, clinical data, conventional and nonconventional CV risk factors, concentrations of adipokines, ultrasonographic measurements and medications of 192 RA patients are summarized in Table 1. The CCA RI, the CCA IMT and concentrations of adipokines did not differ between patients who received biologics and those who did not.

**Table 1:** **Characteristics of patients with rheumatoid arthritis (*n*** **= 192)**

Age (years)	55 (45.3 to 62)
Women	176 (91.7)
Disease duration (years)	9 (4 to 15)
ESR (mm/hour)	35 (20 to 50)
CRP (mg/l)	1.7 (0.5 to 5.5)
DAS28	3.1 (2.5 to 4.1)
TNFα (pg/ml)	3.8 (2.7 to 5.3)
IL-6 (pg/ml)	2.3 (1.7 to 5.5)
Adiponectin (μg/ml)	192 (136.1 to 293.7)
Leptin (ng/ml)	7.7 (3.7 to 13.5)
L:A ratio × 10^5^	3.64 (1.55 to 8.65)
Resistin (ng/ml)	4.7 (2.4 to 8.3)
Metabolic syndrome features	
Metabolic syndrome	27 (14.1)
Waist circumference (cm)	77 (72 to 82.4)
Glucose (mg/dl)	86 (81 to 91)
Triglycerides (mg/dl)	94 (70 to 122)
HDL cholesterol (mg/l)	56 (47 to 65.8)
SBP (mmHg)	124 (114 to 134)
Hypertension	70 (36.5)
HOMA-IR	1.46 (0.96 to 2.27)
Nonmetabolic cardiovascular risk factors	
BMI (kg/m^2^)	22.3 (20.2 to 24.6)
Total cholesterol (mg/dl)	184 (163 to 209.8)
LDL cholesterol (mg/dl)	107.2 (91.2 to 129.4)
Smoking history	19 (9.9)
Mean CCA RI	0.63 (0.60 to 0.66)
Mean CCA IMT (mm)	0.59 (0.51 to 0.68)
Total plaque number	2 (1 to 3)
Total plaque volume (cm^3^)	0.02 (0 to 0.09)
Medications	
Methotrexate	189 (98.4)
Leflunomide	125 (65.1)
Sulfasalazine	135 (70.3)
Hydroxychloroquine	92 (47.9)
Biologics^a^	43 (22.3)

Values are median (interquartile range) or *n* (%).

BMI, body mass index; CCA, common carotid artery; CRP, C-reactive protein; DAS28, Disease Activity Score in 28 joints; ESR, erythrocyte sedimentation rate; HDL, high-density lipoprotein; HOMA-IR, homeostatic model assessment for insulin resistance; IL, interleukin; IMT, intima-media thickness; L:A ratio, leptin:adiponectin ratio; LDL, low-density lipoprotein; RA, rheumatoid arthritis; RI, resistive index; SBP, systolic blood pressure; TNFα, tumor necrosis factor alpha. ^a^Biologics included etanercept, adalimumab and infliximab.

### Association of CCA resistance index with CCA intima-media thickness, and plaque

Significant correlations between CCA RI and CCA IMT and the plaque volume are shown in Table 2. The CCA RI was associated with the mean CCA IMT (*r* = 0.193; *P* = 0.007) and the maximum CCA IMT (*r* = 0.164; *P* = 0.023). The RI was also associated with carotid plaque volume (*r* = 0.167; *P* = 0.021). These correlations remained significant after adjustment for conventional CV risk factors.

**Table 2:** Correlation between CCA resistance index with CCA intima-media thickness and plaque volume

	***r***	***P *** **value**
Mean CCA intima-media thickness	0.193	0.007*
Maximum CCA intima-media thickness	0.164	0.023*
Total plaque volume	0.167	0.021*

CCA, common carotid artery. *P <0.05 after adjustment for conventional cardiovascular risk factors including sex, body mass index, smoking, systolic blood pressure, triglycerides, high-density lipoprotein cholesterol, and low-density lipoprotein cholesterol.

### Association of adipokines with inflammation

Correlations between adipokines and parameters of inflammation are shown in Table 3. Resistin was associated with the ESR (*r* = 0.322; *P <*0.001), CRP (*r* = 0.209; *P* = 0*.*004) and increased disease duration (*r* = 0.176; *P =* 0.014). IL-6 was correlated with the ESR (*r* = 0.450; *P <*0.001), CRP (*r* = 0.468; *P <*0*.*004) and DAS28 ESR (*r* = 0.448; *P <*0.001). Adiponectin was positively correlated with ESR (*r* = 0.162; *P* = 0.025). The associations between resitin and ESR and between IL-6 and ESR and CRP remained significant after adjustment for waist circumference and BMI. Tumor necrosis factor alpha was positively correlated with leptin (*r* = 0.234; *P* = 0.001), resistin (*r* = 0.142; *P* = 0.049) and leptin:adiponectin ratio (L:A ratio) (*r* = 0.226; *P* = 0.002). Adiponectin had a positive association with resistin (*r* = 0.213; *P* = 0.003).

**Table 3:** Coefficients of correlation between adipokines and parameters of inflammation

	**L:A ratio**	**Adiponectin**	**Leptin**	**Resistin**	**TNFα**	**IL-6**
ESR	-0.074	0.162*	-0.005	0.322*^†^	0.181*	0.450*^†^
C-reactive protein	-0.041	0.137	0.026	0.209*	0.087	0.468*^†^
DAS28 ESR	0.021	0.068	0.054	0.095	0.074	0.448*
Disease duration	-0.033	0.094	-0.008	0.176*	0.101	0.134

DAS28, Disease Activity Score in 28 joints; ESR, erythrocyte sedimentation rate; IL, interleukin; L:A ratio, leptin:adiponectin ratio; TNFα, tumor necrosis factor alpha.*P <0.05. ^†^*P* <0.05 after adjustment for adiposity including waist circumference and body mass index.

### Association of adipokines with metabolic risk factors

**Figure 1:**
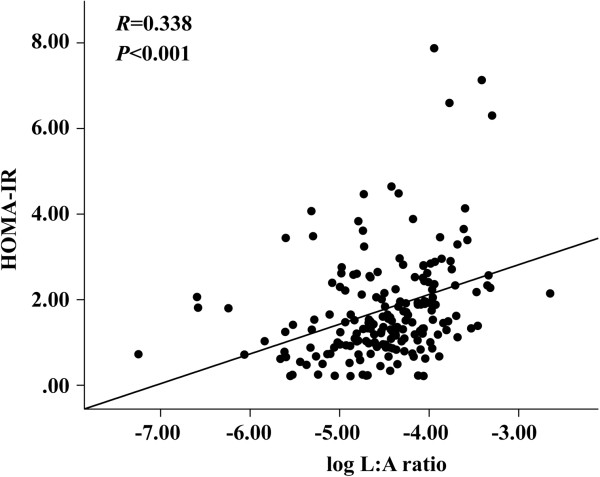
**Correlation of the leptin:adiponectin ratio and homeostatic model assessment for insulin resistance.** Log leptin:adiponectin ratio (L:A ratio) showed highly significant correlation with homeostatic model assessment for insulin resistance (HOMA-IR) (*r* = 0.338; *P <*0.001), and the correlation remained significant after adjusting conventional cardiovascular risk factors.

Correlations between adipokines and metabolic risk factors are shown in Table 4. HOMA-IR, metabolic syndrome criteria numbers, waist circumference, systolic blood pressure, glucose, TG, and HDL cholesterol were included in the metabolic risk factors. Leptin and the L:A ratio were associated with multiple metabolic risk factors. Leptin was associated with HOMA-IR (*r* = 0.369; *P <*0.001), Metabolic syndrome criteria number (*r* = 0.388; *P <*0.001), waist circumference (*r* = 0.488; *P <*0.001), and TG (*r* = 0.264; *P <*0.001). The L:A ratio was correlated with HOMA-IR (*r* = 0.377; *P <*0.001), Metabolic syndrome criteria number (*r* = 0.402; *P <*0.001), waist circumference (*r* = 0.431; *P <*0.001), systolic blood pressure (*r* = 0.164; *P =* 0.023), TG (*r* = 0.241; *P =* 0.001) and HDL cholesterol (*r* = -0.169; *P* = 0.019). The L:A ratio showed a significant correlation with HOMA-IR (Figure 1), and the correlation remained significant after adjusting for conventional CV risk factors.

**Table 4:** Coefficients of correlation between adipokines and parameters related to insulin resistance

	**L:A ratio**	**Adiponectin**	**Leptin**	**Resistin**	**TNFα**	**IL-6**
HOMA-IR	0.377*^†^	-0.113	0.369*	0.093	0.204*	0.076
MetSyn criteria number	0.402*	-0.159*	0.388*	0.011	0.228*	-0.016
Waist circumference	0.431*	-0.094	0.488*	-0.037	0.239*	0.045
SBP	0.164*	-0.068	0.119	0.033	0.112	-0.001
Glucose	0.001	-0.124	-0.076	-0.116	0.082	-0.102
Triglycerides	0.241*	-0.102	0.264*	0.098	0.190*	-0.073
HDL cholesterol	-0.169*	0.261*	-0.011	-0.002	-0.189*	-0.025

HDL, high-density lipoprotein; HOMA-IR, homeostatic model assessment for insulin resistance; IL, interleukin; L:A ratio, leptin:adiponectin ratio; MetSyn, metabolic syndrome; SBP, systolic blood pressure; TNFα, tumor necrosis factor alpha. *P <0.05. ^†^*P* <0.05 after adjustment for conventional cardiovascular risk factors including age, sex, smoking, systolic blood pressure, waist circumference, glucose, triglycerides, HDL cholesterol, and low-density lipoprotein cholesterol.

### Associations of common carotid resistance index with L:A ratio after adjustment for conventional and nonconventional cardiovascular risk factors

Age, waist circumference, BMI, HOMA-IR, and L:A ratio were associated with increased CCA RI in the univariate linear regression, as shown in Table 5. The L:A ratio was significantly associated with CCA RI after adjustment for age, waist circumference, BMI and HOMA-IR (β = 47.996 ± 22.723, *r* = 0.036) (data not shown). The L:A ratio remained significantly associated with CCA RI after adjusting for conventional CV risk factors, and for nonconventional CV risk factors including HOMA-IR, ESR and CRP (β = 49.558 ± 22.613; *P* = 0.030), as shown in Table 5.

**Table 5:** Linear regression analysis for associations of CCA resistance index with dependent variables

**Variable**	**Univariate linear regression analysis**			**Multivariate linear regression analysis**^**a**^			
	**β**	**SE (β)**	***P *** **value**	**β**	**SE (β)**	***P *** **value**	**Final model (*R***^**2**^**)**
L:A ratio	66.661	22.157	0.003	49.558	22.613	0.030	0.210
HOMA-IR	0.008	0.003	0.012	0.003	0.003	0.381	
Age	0.001	0.000	0.001	0.001	0.000	0.000	
Waist circumference	0.002	0.000	0.000	0.001	0.001	0.064	
BMI	0.004	0.001	0.003	0.001	0.002	0.643	

BMI, body mass index; CCA, common carotid artery; HOMA-IR, homeostatic model assessment for insulin resistance; L:A ratio, leptin:adiponectin ratio; SE, standard error. ^a^Multiple linear regression analysis for L:A ratio, conventional cardiovascular risk factors (age, sex, smoking, systolic blood pressure, waist circumference, BMI, glucose, triglycerides, high-density lipoprotein cholesterol, low-density lipoprotein cholesterol, HOMA-IR), and nonconventional cardiovascular risk factors (erythrocyte sedimentation rate and C-reactive protein) and L:A ratio.

## Discussion

Our study showed a clear relationship between the L:A ratio and CCA RI in patients with RA. We found that CCA RI was correlated with CCA IMT and total plaque volume in RA. Among adipokines, resistin and IL-6 were correlated with inflammatory parameters. Leptin and the L:A ratio were related to metabolic risk factors, including HOMA-IR. The L:A ratio was associated with CCA RI independent of conventional CV risk factors and nonconventional risk factors, including HOMA-IR, ESR and CRP.

The RI is a functional parameter that is easily determined by Doppler sonography in contrast to IMT, a morphological parameter [16]. Before the appearance of morphological alterations that are detectable by the thickening of the intima-media complex, the early form of arteriosclerosis leads to endothelial dysfunction as an exclusively functional disorder characterized by reduced elasticity and increased peripheral resistance [17]. The RI may thus detect the atherosclerotic process as early as observed by IMT. A previous study revealed that the carotid RI predicted the atherosclerosis score and CV mortality and morbidity comparable with the IMT [17]. CV risk factors were independently associated with the carotid RI and IMT [18]. However, the carotid RI had not been previously demonstrated in RA cohorts; this is the first study to measure the carotid RI in RA patients, to our knowledge. In our study, CCA RI was associated with CCA IMT, the estimated total plaque volume independent of conventional CV risk factors. Carotid RI might serve as a marker (complementary to IMT) to signal early atherosclerosis in RA. Further studies will be needed to validate the value of the RI in RA patients.

Leptin and adiponectin are the most abundant adipokines produced by adipocytes. These adipokines are thought to provide an important link between obesity, insulin resistance and related inflammatory disorders [11]. Leptin is known to have proinflammatory and proatherogenic activities [19,20]. Leptin concentrations were independently associated with coronary heart disease [21] and predicted CV events in subjects with coronary atherosclerosis [22]. In contrast to leptin, adiponectin is a protein with insulin-sensitizing properties and anti-inflammatory effect [23,24]. Clinical studies identified an association between low serum concentration of adiponectin and CV events [25,26]. Resistin has proinflammatory properties in human [20]. Although studies in animal models consistently show that resistin induces insulin resistance [27], evidence for this effect in humans is less clear. We showed that concentrations of adipokines were associated with systemic inflammation in this study. Resistin and IL-6 were associated with inflammatory markers including the ESR, CRP, DAS28 ESR (only with IL-6) and disease duration (only with resistin). This was consistent with previous reports of serum resistin and IL-6 being related to systemic inflammation in RA [28-30]. Meanwhile, leptin and adiponectin did not show any associations with inflammatory parameters. This lack of association might be because of relatively low inflammatory burdens of subjects in this study. In addition, adiponectin was positively correlated with resistin. This finding might be due to the compensatory mechanism of adiponectin in the presence of inflammation in RA. Our results supported a recent report that suggested a compensatory increase of adiponectin receptors to counteract the excess inflammatory and atherogenic process in coronary artery disease [31].

In the present study, leptin and the L:A ratio were related to metabolic risk factors and HOMA-IR, consistent with findings that leptin resistance occurred and adiponectin decreased in the insulin-resistant status [32]. Rho and colleagues showed only leptin as being associated with HOMA-IR in RA patients. However, the L:A ratio was reported to be correlated with insulin resistance and to possibly predict the risk of metabolic syndrome in the nondiabetic population [33,34], which might be due to a reciprocal response of leptin and adiponectin to increasing adiposity. Consistent with this, we confirmed an association between the L:A ratio and metabolic risk factors, especially HOMA-IR in patients with RA.

Additionally, the L:A ratio was related to carotid IMT, and was an independent factor for predicting CV disease in healthy population [35]. Meanwhile, only resistin and IL-6 were related to endothelial activation in RA [36,37], but leptin and adiponectin alone were not related to carotid IMT in patients with RA [38]. We therefore investigated the association between the L:A ratio and carotid atherosclerosis in patients with RA, and revealed that the L:A ratio was independently correlated with the CCA RI, after adjusting for conventional risk factors and nonconventional risk factors, including HOMA-IR. The L:A ratio might be an independent factor for the prediction of CV risk in RA. There are few reports available on the L:A ratio and CV risk in other inflammatory diseases. In patients who were on peritoneal dialysis for end-stage renal disease, the L:A ratio has been reported as being related to mortality secondary to chronic inflammation [39]. The L:A ratio being independently related to CV risk in RA is a novel finding.

Little is known about the relationship between adipokines, insulin resistance, and atherosclerosis in patients with RA. Circulating proinflammatory cytokines that secreted from inflamed joints may alter the function of distant tissues (for example, adipose, skeletal muscle, liver, and vascular endothelium) to generate a spectrum of proatherogenic changes that include insulin resistance, prothrombotic effects and endothelial dysfunction [40]. We focused on insulin resistance, one of the pathways that contribute to atherosclerosis in RA, and had hypothesized that adipokines link inflammation, insulin resistance, and carotid atherosclerosis. Insulin resistance maintains some degree of inflammation, and RA itself exhibits persistent inflammation, and both insulin resistance and RA continue to affect CV risk, thus mutually reinforcing each other [41]. This reinforcement could be one mechanism underlying the pathogenesis of accelerated atherosclerosis in RA, and our results support this hypothesis.

## Conclusions

We investigated the overall relationship between adipokines, inflammation, insulin resistance, and the carotid RI in patients with RA. The L:A ratio was significantly associated with HOMA-IR and with carotid RI. Finally, the L:A ratio might be an independent factor for the prediction of CV risk in RA.

## Abbreviations

BMI: Body mass index; CCA: Common carotid artery; CRP: C-reactive protein; CV: Cardiovascular; DAS28: Disease Activity Score in 28 joints; ESR: Erythrocyte sedimentation rate; HDL: High-density lipoprotein; HOMA-IR: Homeostatic model assessment for insulin resistance; IL: Interleukin; IMT: Intima-media thickness; L:A ratio: Leptin:adiponectin ratio; RA: Rheumatoid arthritis; RI: Resistive index; TG: Triglycerides.

## Competing interests

The authors declare that they have no competing interests.

## Authors’ contributions

All authors were involved in drafting or critically reviewing the manuscript. YK and Y-BP participated in the study conception and design. YK, H-JP, S-WL, S-KL, and Y-BP participated in the acquisition of data. YK, H-JP, M-IK, H-SL, S-WL, S-KL, and Y-BP contributed to the analysis and interpretation of data. All authors read and approved the manuscript for publication.
